# Accelerating Inference of Convolutional Neural Networks Using In-memory Computing

**DOI:** 10.3389/fncom.2021.674154

**Published:** 2021-08-03

**Authors:** Martino Dazzi, Abu Sebastian, Luca Benini, Evangelos Eleftheriou

**Affiliations:** ^1^IBM Research Europe, Rüschlikon, Zurich, Switzerland; ^2^Eidgenössische Technische Hochschule Zürich, Zurich, Switzerland

**Keywords:** convolutional neural network, in-memory computing, computational memory, AI hardware, neural network acceleration

## Abstract

In-memory computing (IMC) is a non-von Neumann paradigm that has recently established itself as a promising approach for energy-efficient, high throughput hardware for deep learning applications. One prominent application of IMC is that of performing matrix-vector multiplication in O(1) time complexity by mapping the synaptic weights of a neural-network layer to the devices of an IMC core. However, because of the significantly different pattern of execution compared to previous computational paradigms, IMC requires a rethinking of the architectural design choices made when designing deep-learning hardware. In this work, we focus on application-specific, IMC hardware for inference of Convolution Neural Networks (CNNs), and provide methodologies for implementing the various architectural components of the IMC core. Specifically, we present methods for mapping synaptic weights and activations on the memory structures and give evidence of the various trade-offs therein, such as the one between on-chip memory requirements and execution latency. Lastly, we show how to employ these methods to implement a pipelined dataflow that offers throughput and latency beyond state-of-the-art for image classification tasks.

## 1. Introduction

In recent years, Deep Neural Networks (DNNs) have revolutionized the field of Machine Learning by reaching unprecedented accuracy in a large number of cognitive data analysis tasks. DNNs are currently being used in a wide variety of applications, ranging from image classification (He et al., 2016) to autonomous driving (Bojarski et al., 2016) and natural language interpretation (Vaswani et al., 2017). As the applications increase in number and complexity, so do DNN architectures, and along with them the hardware architectures for their execution and training.

Historically, DNNs run on general purpose processors, such as CPUs and GPUs. While this solution is still widely employed and GPUs are constantly improving their metrics of energy consumption and execution time for training and inferencing, they are inadequate for application areas with power envelopes of sub Watt or of very few Watts, which are generally referred to as the IoT or edge computing realm. To this end, custom hardware platforms such as application-specific integrated circuits (ASICs) are being designed (Chen et al., 2016) for low power and efficient execution of DNNs. ASICs are quite energy efficient in terms of TOPS/W and can reach state-of-the art accuracy and throughput in a variety of tasks (Jouppi et al., 2017), albeit at the expense of time consuming circuit design and, to a certain extent, limited scope of execution. Moreover, a hardware-aware training of the DNNs (Han et al., 2015; He et al., 2017; Jacob et al., 2018) is often needed.

Independently from the hardware platform, be it general purpose processors or ASICs, different performance can be obtained on the basis of the computational paradigm being used. In general, von Neumann architectures are inherently limited in performance by the need to move data from the memory to the computational units: in modern DNN models, with a parameter count that can reach hundreds of millions (Huang et al., 2017; Vaswani et al., 2017) retrieving them from a memory can severely hinder performance. This phenomenon, also known as the von Neumann bottleneck, has led to many research efforts aiming at alternative, non-von Neumann paradigms. Among these, we look in depth into IMC (Prezioso et al., 2015; Burr et al., 2017; Hu et al., 2018; Ielmini and Wong, 2018; Le Gallo et al., 2018; Xia and Yang, 2019; Sebastian et al., 2020), a computational paradigm showing promise for unprecedented performance and energy efficiency, targeting specifically the main computational load of DNNs: matrix-vector multiplications. With IMC, we take advantage of a set of resistance-based or charge-based memory devices, such as memristive (Sebastian et al., 2019; Joshi et al., 2020) or CMOS-based devices (Valavi et al., 2019). By organizing these devices in a crossbar array configuration, based on their physical properties, a matrix-vector multiplication can be carried out with O(1) time complexity, contrary to the $O\left(N^{2}\right)$ time complexity of this operation on traditional architectures. However, in order to fully exploit this new computing paradigm, in the design of IMC-based hardware we must rethink well-established architectural choices and provide novel methodologies for optimizing the dataflow. Specifically, while the number and size of synaptic weights can vary greatly within the layers of a DNN, the IMC crossbar arrays on which they are mapped have pre-determined, fixed shapes. Consequently, the mapping of synaptic weights is a pivotal problem to optimize in order to fully exploit the potential of IMC in DNN applications.

Moreover, while IMC obviates the need to communicate synaptic weights, the intermediate results must be cached on the local memories of the IMC cores. Also in this case, new approaches must be developed for handling the data efficiently and according to the dataflow.

In this work, we focus on the problem of executing image classification tasks on IMC-based hardware architectures. Specifically, we focus on Convolutional Neural Networks (CNNs), which represent the state-of-the-art for a variety of image processing applications. In section 2, we propose a novel IMC core architecture for inference of CNNs. Moreover, we present novel methods for mapping weights on the IMC crossbar array and activations on the local memory of the IMC core. These methods enable high-throughput, efficient execution of CNNs on the IMC hardware. Note that while these contributions are presented as different parts that organically belong to a IMC-based accelerator for inference of CNNs, the methodologies and approaches developed here, have universal applicability regardless of the overall architectural configuration. In section 3, we introduce the dataflow of the IMC-based accelerator and present as a case study the execution of ResNet-32 on the CIFAR-10 dataset. This section gives evidence of how our proposed methodologies applied to IMC yield beyond state-of-the-art performance compared to non-IMC ASICs targeting the same dataset. Lastly, section 4, compares our approach with previous works in the field and concludes the paper.

## 2. Methods

### 2.1. Hardware Architecture Overview

We identify the In-Memory Computing core (IMC core) as the unit building block of the IMC hardware architecture. The IMC core is built around a crossbar array executing the matrix-vector multiplication and features peripheral circuitry for the additional functionality required by CNNs.

**Figure 1:**
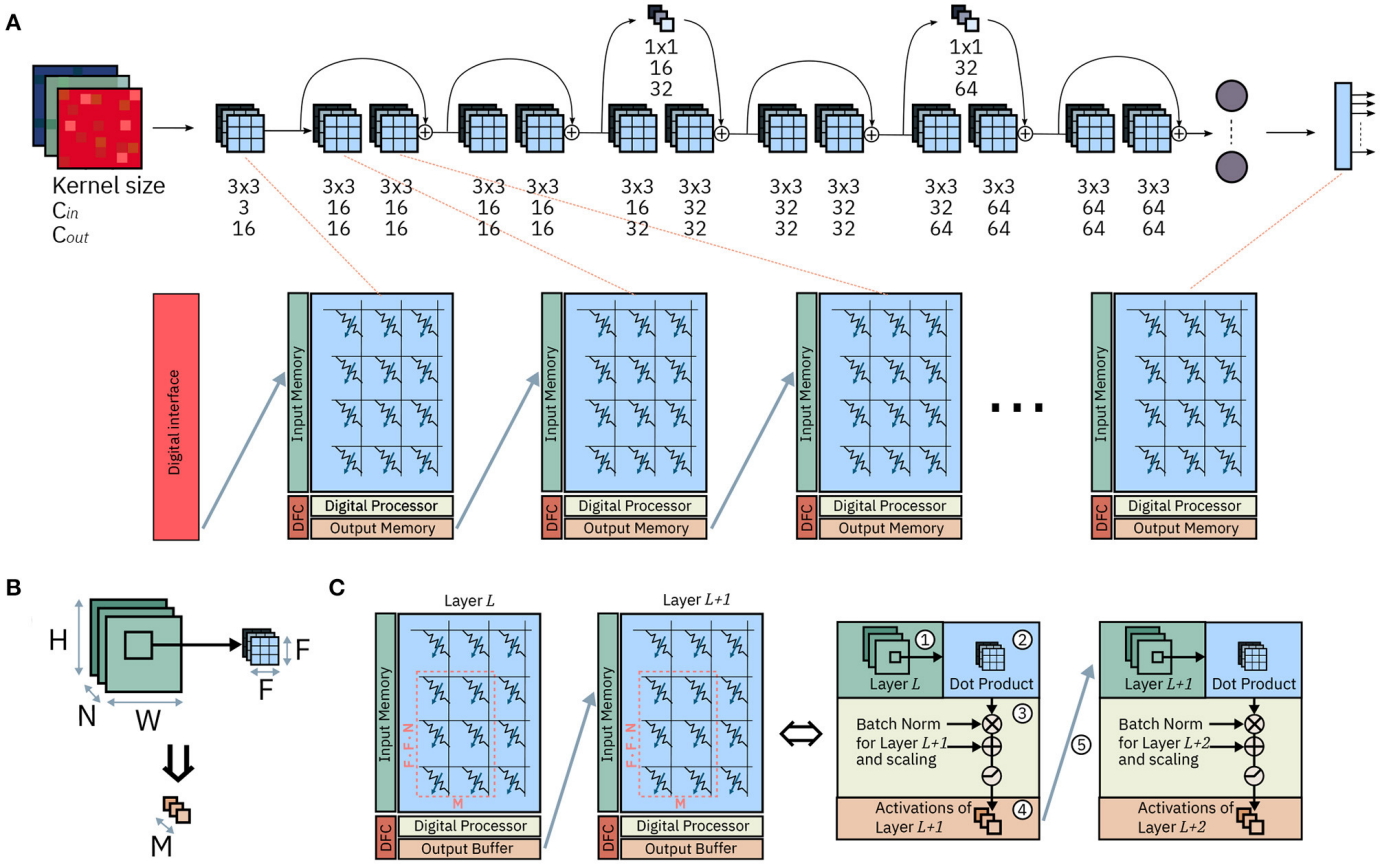
Mapping of ResNet-32 on an array of IMC cores. **(A)** Mapping of the network on the array: each layer is mapped onto one IMC core. The synaptic weights are mapped on the computational memory elements, while operations on pre-activations or activations are carried out by the Digital Processor. **(B)** One matrix-vector multiplication for a convolutional layer. **(C)** Dataflow between two IMC cores; the dataflow is scheduled by the Dataflow Controller. The system level representation (left) and, color-coded, the corresponding functionality of each component (right).

Figure 1 shows the mapping of the CNN layers on the IMC cores and the tasks carried out during execution by each component. We start by describing the operation required by CNNs in order to understand how these are employed on the hardware architecture and how the hardware itself is designed in order to facilitate their execution.

A CNN architecture is typically comprised of a series of interconnected layers, each layer defined by learnable parameters and typically one activation function. The result of the activation function is referred to as activation. As the name suggests, every layer of a CNN performs a convolution on the activations. A convolutional layer transforms an input volume of feature maps of size *H*_*in*_ · *W*_*in*_ · *C*_*in*_ into an output volume of feature maps of size *H*_*out*_ · *W*_*out*_ · *C*_*out*_. Unless specified, we will consider in our study input and output volumes with *H*_*in*_ = *H*_*out*_ = *H* and *W*_*in*_ = *W*_*out*_ = *W*. Note that these assumptions do not affect generality, since for the layers in which pooling, striding, and padding are involved, the approach we follow in designing the various architectural choices would remain the same. Also, we will refer to the activations along the plane marked by *H* and *W* as lying on the (*H, W*) plane.

Each convolutional layer consists of many dot products. Specifically, every element of the output volume in the (*H, W*) plane is calculated as the dot product between a patch of the input volume of size *F*_1_ · *F*_2_ · *C*_*in*_ with an equally sized matrix of parameters of that layer, also called kernel weights. The dot product is executed *C*_*out*_ times by applying *C*_*out*_ different kernel weights in order to obtain all *C*_*out*_ output channels of the output volume. The results of the matrix-vector multiplication are called pre-activations. In matrix form, the matrix-vector multiplication for one pre-activation at layer *L* + 1 across all channels at a generic position (*u, v*) is expressed by Equation (1), where $\alpha_{i,j}^{n,L}$ indicates an activation at layer *L* and position *(i, j)* at channel *n*, $\gamma_{i,j}^{n,L}$ denotes a pre-activation layer *L*, position (*i, j*) and channel *n*, and ***K*** represents the kernel matrix. One element of the kernel matrix for a position *(a,b)*, input channel *v*, and output channel *w* is indicated as $k_{a,b}^{v,w}$. We consider *C*_*in*_ = *N*, *C*_*out*_ = *M*, and *F*_1_ = *F*_2_ = *F*.

(1) $$\begin{array}{l} \gamma_{u,v}^{L+1}=\alpha_{i:i+F-1,j:j+F-1}^{L}\cdot \\ \;K={\left[\begin{array}{c} \alpha_{i,j}^{0,L}\\ \ldots \\ \alpha_{i+F-1,j+F-1}^{N/2-1,L}\\ \alpha_{i,j}^{N/2,L}\\ \ldots \\ \alpha_{i+F-1,j+F-1}^{N-1,L} \end{array}\right]}^{T}\left[\begin{array}{ccc} k_{0,0}^{0,0} & \ldots & k_{0,0}^{0,M}\\ \ldots & \ldots & \ldots \\ k_{F-1,F-1}^{N/2-1,0} & \ldots & k_{F-1,F-1}^{N/2-1,M}\\ k_{0,0}^{N/2,0} & \ldots & k_{0,0}^{N/2,M}\\ \ldots & \ldots & \ldots \\ k_{F-1,F-1}^{N,0} & \ldots & k_{F-1,F-1}^{N,M} \end{array}\right] \end{array}$$

Subsequently to the operation on the kernel weights, the resulting pre-activations undergo a post processing that typically involves additive and multiplicative scalings, known as batch normalization (Ioffe and Szegedy, 2015) and a non-linear activation function. Given the operations described above, the matrix-vector multiplication that transforms activations from one layer into pre-activations of another is particularly suited for IMC, while the subsequent operations on pre-activations are better suited for standard digital processing units.

Figure 1A shows the mapping of a CNN, ResNet-32, on an array on IMC cores. The network is mapped on the physical array so that at most one layer is mapped on each IMC core. In the cases in which the size of the layer is greater than that of the IMC core, the synaptic weights of the layer are split between multiple IMC cores. The topology of the network is mapped on the IMC cores, implemented as crossbar arrays, so that edges of the dataflow graph of the CNN correspond to communication channels in the physical array. While the design of the communication fabric for such an architecture is not the subject of this work, we will assume that any IMC core can communicate in one timestep to any other IMC core with which communication is required, as presented in Dazzi et al. (2019). Figure 1B shows figuratively the matrix-vector multiplication of Equation 1 for a convolutional layer where *F*_1_ = *F*_2_ = *F*. A subset of the input volume (in green) is multiplied by the kernel weights (in blue); after batch normalization and execution of the activation functions, the results are the output activations across all output channels *C*_*out*_ and one position on the *H, W* dimensions (in orange). Figure 1C shows the hardware architecture of two successive IMC cores and, color coded, the role of every component in the execution of the CNN. The IMC core consists of the Crossbar Array, an Input Memory, a Digital Processor, an Output Memory and a Dataflow Controller.

The Input Memory stores the input activations of one layer, which during execution are either received from an input scratchpad or from another IMC core executing another layer. When needed for computational purposes, the activations are read from the Input Memory and provided to the interface of crossbar array ①. The crossbar array maps the kernel weights of one layer to its IMC devices and executes the matrix-vector multiplication between a patch of the input volume and the kernel weights themselves expressed in Equation (1) ②. The result of this computation are *C*_*out*_ pre-activations, i.e., the results of one matrix-vector multiplication before any post-processing and application of the non-linear activation function. The Digital Processor executes the remainder of the computation that transforms pre-activations into activations ③. Namely, it takes care of all computations in CNNs not adequate for IMC. In our example, these include batch normalization and the application of activation functions. Lastly, the output memory collects the output activations that are the result of the computation ④; These results are then delivered, via communication links, to the next layers (cores) for further processing ⑤.

**Figure 2:**
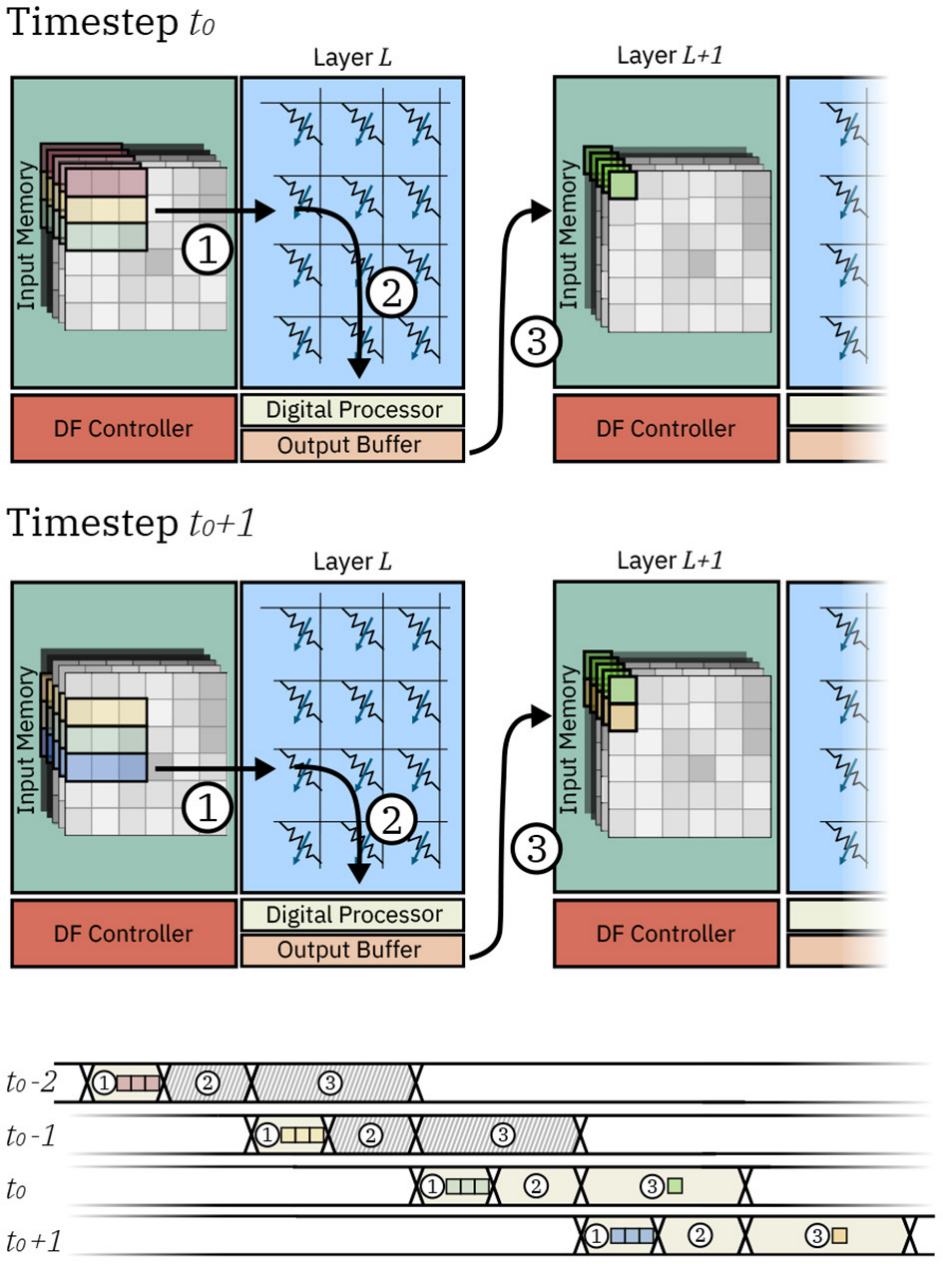
Data movement in two IMC cores at two consecutive timesteps *t*_0_ and *t*_0_ + 1. Execution steps are represented as (1) fetching and loading of the data, (2) computation, and (3) communication of the pixel to other IMC cores. The timechart shows the execution steps for the IMC core executing layer *L*. At timesteps *t*_0_ − 2 and *t*_0_ − 1 input pixels are loaded on the crossbar array. As data is not yet sufficient for the computation of any pixel, no computation or communication takes place. At timestep *t*_0_, sufficient pixels are available to compute on the crossbar array and one input pixel is computed and communicated to other IMC cores. Likewise at timestep *t*_0_ + 1, a new row of pixels is loaded and the IMC core executed the computation and communication of another output pixel.

Figure 2 shows the detailed dataflow of two IMC cores executing two subsequent layers *L* and *L+1* and the dataflow chart for the IMC core executing layer *L*. In the example, we assume a 3x3 kernel and no padding. Firstly, rows of input pixels to layer *L* are fetched from the Input Memory and loaded to the Crossbar Array ①. In this phase, the pixels are fetched from the local SRAM and loaded on the local buffers of the Crossbar Array, which temporarily store pixels for the subsequent computation to be executed. The operation is repeated for timesteps *t*_0_ − 2 (red row of pixels), *t*_0_ − 1 (yellow row of pixels), and *t*_0_ (green row of pixels), after which enough data for one dot product has been loaded to the buffers of the Crossbar Array. Consequently, the computation in the crossbar array ② can take place at timestep *t*_0_. Finally, timestep *t*_0_ concludes with the delivery of the pixel that was computed to the local memory of the subsequent core ③. In can be seen how, aside from the initial loading of pixels at timesteps *t*_0_ − 2 and *t*_0_ − 1, the IMC core assigned to layer *L* requires a new row of pixels to be fetched for computation. Indeed, at timestep *t*_0_ + 1, one new row of pixels (in blue) is loaded, followed by computation of one dot product and delivery of the pixel to the IMC core assigned to layer *L+1*.

### 2.2. Mapping of Weights

**Figure 3:**
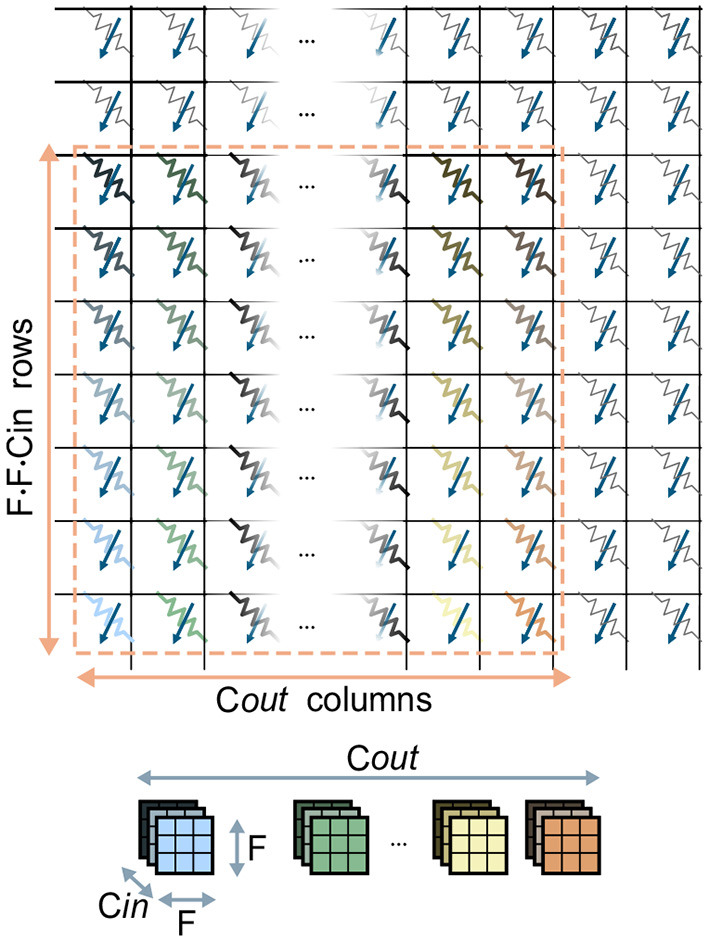
Mapping of one kernel matrix of size *F* · *F* · *C*_*in*_ · *C*_*out*_ on a crossbar array. The kernel weights **(top)** are color coded to reflect their placement on the devices **(bottom)**. The *F* · *F* · *C*_*in*_ kernel weights responsible for one of the *C*_*out*_ output channels are mapped on one column of the array.

In CNNs, the synaptic weights, also referred to as kernel weights and responsible for the matrix-vector multiplications, comprise a convolutional layer. In this section, we will refer to the overall matrix of kernel weights of size *F*_1_ · *F*_2_ · *C*_*in*_ × *C*_*out*_ as the kernel matrix. Also, for the sake of brevity, we will refer to the activations for one position in the (*H, W*) plane across all channels as one *pixel*. Figure 3 shows the mapping of one kernel matrix on a crossbar array. Without loss of generality, it is assumed that one kernel weight is mappable on one computational memory device. Note that this is a reasonable assumption given the common precision requirements of weights in DNNs (Joshi et al., 2020) and the effective precision offered by the memory devices used for IMC. Our approach can however be easily extended to cases where a single weight is mapped on multiple devices. According to this mapping approach, the kernel matrix is unrolled so that the kernel weights associated to one output channel are placed along one column of the crossbar. In this way, the *C*_*out*_ columns of the kernel matrix are mapped on the same amount of adjacent columns of the crossbar array. Based on this physical placement, one matrix-vector multiplication is performed as follows: first, the input data-patch, retrieved from the Input Memory, is unrolled and provided at the input rows of the crossbar, matching the corresponding rows of the kernel matrix. Then, from each column, the pre-activation for a single channel is read back and converted to a digital value. The pre-activations will subsequently undergo digital processing, which in principle implements the non-linear function and any other digital processing that is required.

Fundamentally, the problem of mapping a kernel matrix to a crossbar array constitutes the mapping of a shape of (*F*_1_ · *F*_2_ · *C*_*in*_) × *C*_*out*_ elements onto a grid of fixed size composed of computational memory elements. Although the *F*_1_, *F*_2_, *C*_*in*_, *C*_*out*_ parameters change from layer to layer and from network to network, state-of-the-art CNNs for image classification (He et al., 2016; Huang et al., 2017) typically feature kernels of size *F*_1_ = *F*_2_ = 3, and channel sizes in powers of two, commonly ranging from 16 to 1024. Furthermore, usually *C*_*in*_ = *C*_*out*_ for the majority of the internal layer of the CNNs (Krizhevsky et al., 2012; He et al., 2016). In light of this assessment, we will focus on the case where *F*_1_ = *F*_2_ = 3 and *C*_*in*_ = *C*_*out*_, as this is the set of hyperparameters which is the most common within the architectures of the CNNs we take into consideration. Regarding the dimensionality of the crossbar arrays, it appears that practical implementations adopt square sizes, primarily for generality and for the possibility of performing the reverse read operation in the case the array was designed also for training DNNs (Nandakumar et al., 2018). Based on these considerations, the kernel matrices typically appear to have an aspect ratio equal to *F*_1_ · *F*_2_ = 9 (i.e., they require 9 times more rows than columns), and are mapped on a crossbar array grid with aspect ratio equal to one. This implies that a large number of the crossbar array devices will end up being unmapped, and therefore unutilized, during the execution of CNNs. We try to avoid this issue by proposing two methods of parallelization of the computation of the convolutional layers by replication of the kernel matrix. In these methods, we map multiple replicas of the kernel matrix on the crossbar array for one convolutional layer in order to execute in parallel multiple matrix-vector multiplications of the same layer. Note that the number of kernel replicas mapped on the crossbar array effectively represent the number of dot products that can be executed in parallel on the array. Consequently, the number of kernel replicas is equivalent to the degree of parallelism of the dot products.

#### 2.2.1. Parallelizing Computation Across One Direction

**Figure 4:**
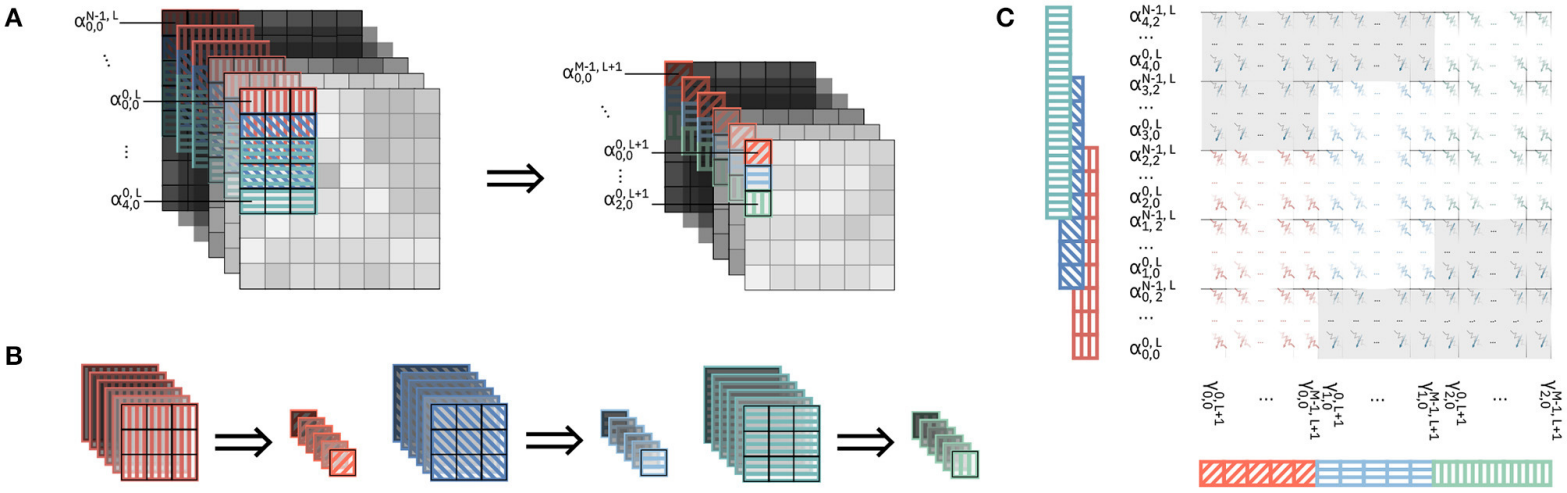
Parallelization of the computation along one direction through mapping of kernel matrix replicas on the crossbar array. In this example, we display the parallelization of three matrix-vector multiplications of the convolutional layer. Devices outside the colored patches are unmapped. **(A)** Input volume (left) and output volume (right) of feature maps for a given layer, where three matrix-vector multiplications are executed in parallel. The patches required for every matrix-vector multiplications are highlighted in the input volume and color-patterned. The resulting output activations in the output volume are color-patterned in a matching color. **(B)** Correspondence between the input patches in **(A)** and the resulting output activation. **(C)** Mapping of the kernel matrix replicas for the three matrix-vector multiplications on the crossbar array. The color of the devices corresponds to the similarly colored input and output activations in **(B)**. Input activations are provided to the crossbar array in one timestep in an order that is highlighted by the same color pattern as in **(A,B)**.

In this method, we parallelize the computation so that the outputs are pre-activations along one direction of the (*H, W*) plane of the output volume. Figure 4 shows the parallelization of three matrix-vector multiplications for a single convolutional layer with a kernel of size *F*_1_ = *F*_2_ = 3.

Figure 4A shows the input and output feature map volumes. In the input volume, we highlight the input patches required for each of the matrix-vector multiplications we parallelize and in the output volume we highlight the positions of the resulting pre-activations. All the activations from the various input patches will be provided in parallel to the rows of a crossbar array, and consequently all the pre-activations of the output volume will be calculated in parallel on the columns of the crossbar array. Figure 4B shows the single input patches of the input volume, colored and patterned, and the corresponding pre-activations on the output volume with the same kernel matrix. It can be noted in Figure 4A that, while each of the three input patches comprise 27·*C*_*in*_ activations, they overlap and overall comprise (5 × 3) · *C*_*in*_ unique activations. Figure 4C shows the mapping of the kernel replicas on the crossbar array. Three replicas of the kernel matrix are mapped on the crossbar, each kernel matrix by itself occupying an area of *F*_1_ · *F*_2_ · *C*_*in*_ rows and *C*_*out*_ columns. Different color patterns represent input activations and output pre-activations for different matrix-vector multiplications on the same input volume in Figure 4A. Note that, because of the overlapping in the input patches, some input activations belong to more than one input patch. Specifically, in Figure 4C, input activations with overlapping color patterns represent activations of the input volume that belong to various input patches. Consequently, as the input pixels for different matrix-vector multiplications are shared, some rows of the crossbar are shared between different kernels. Specifically, assuming *F*_1_ = *F*_2_ = *F*, while one kernel matrix occupies a (*F* · *F* · *C*_*in*_) × *C*_*out*_ area on the crossbar, any additional kernel matrix adds *F* · *C*_*in*_ rows and *C*_*out*_ columns.

#### 2.2.2. Parallelizing Computation Across Two Directions

**Figure 5:**
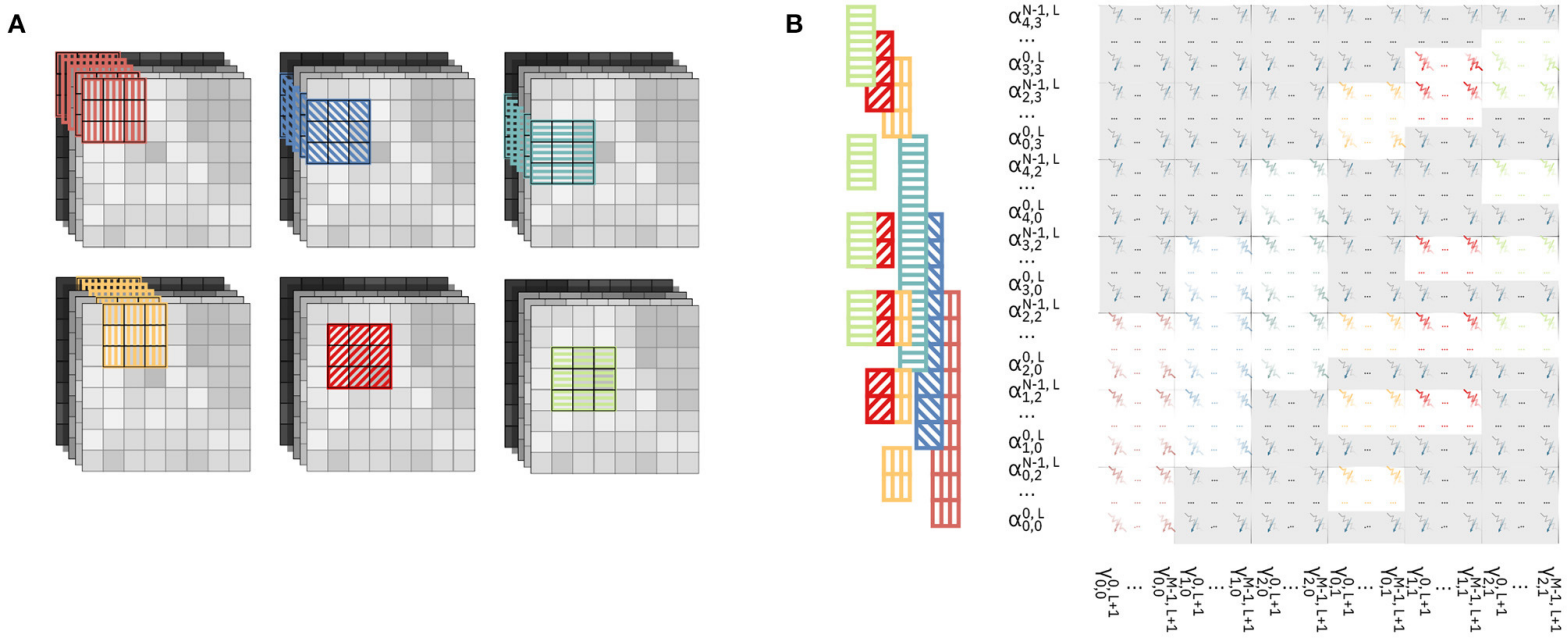
Parallelization of the computation along two directions by mapping of kernel matrix replicas on the crossbar array. **(A)** Color-patterned position of the six input patches for the six matrix-vector multiplications executed in parallel on the crossbar. **(B)** Mapping of the kernel matrix replicas on the crossbar array. Input activations are provided to the crossbar array in an order that is highlighted by the same color pattern as in **(A)**.

In this method, we parallelize the computation so that the output are pre-activations along on both directions of the (*H, W*) plane of the output volume. In Figure 5A, we color-code and pattern the input patches for the parallelized matrix-vector multiplications on the feature maps. We parallelize overall six matrix-vector multiplications, with the input patches covering collectively a 5-by-4 area on the (*H, W*) plane of the input volume. Figure 5B shows the physical mapping of the kernel replicas on the crossbar and the position of the activations provided as inputs. It can be noted that, contrary to the previous method, the sharing of rows among different kernels is more scattered, and generates a less regular pattern of mapping of both activations to the rows of the crossbar and kernel weights to the devices. With this method, for every replica of the kernel matrix mapped onto the crossbar, *C*_*out*_ columns are assigned to the new output to be computed, while the number of additional rows depends on the position of the patch on which the kernel is supposed to perform the computation. In particular, based on the position, the activations required by one kernel replica may or may not have been already in use by rows in the crossbar. For example, the kernel associated with the yellow patch, i.e., the one with a vertical stripe pattern in Figure 5A, requires *F* · *C*_*in*_ rows to be assigned. However, the kernels associated with the red diagonal stripe patterned and light green horizontal stripe patterned patches only require *C*_*in*_ new rows to be assigned for each.

#### 2.2.3. Comparison of Parallelization Methods

Given the many variables in the mapping of kernel matrices to crossbar arrays (e.g., crossbar size, kernel size, and number of input and output channels that might change even within the same CNN), a reasonable *figure of merit* of a mapping scheme should be based on the aspect ratio of the kernel matrices that are mapped on the crossbar. Indeed, assuming crossbars of aspect ratio equal to 1 and given a number of kernel matrices to be mapped on that crossbar, then the greatest number of kernel matrices that can fit on the crossbar is achieved by a method that makes the aspect ratio of the shape of the overall replicas of the kernel matrix closer to 1. In this section, we will refer to the method proposed in section 2.2.1 as Method 1, and the method proposed in section 2.2.2 as Method 2. Also, we will refer to the overall matrix comprising a number of kernel matrix replicas to be mapped onto one crossbar as the collective kernel matrix. In Method 1, the aspect ratio of *n* kernel replicas (collective kernel matrix) mapped on the crossbar can be expressed in closed form and, for *F*_1_ = *F*_2_ = *F*, is equal to

(2) $$\begin{array}{l} A\left(n\right)=F^{2}\cdot \frac{C_{in}}{C_{out}}\cdot \frac{1+\left(n-1\right)\cdot \frac{1}{F}}{1+\left(n-1\right)} \end{array}$$

It can readily be seen that for *C*_*in*_ = *C*_*out*_, then *A*(1) = *F*^2^, and *A*(∞) = *F*. Thus, as the number of kernel matrix replicas increases, the aspect ratio of the collective kernel matrix decreases. Specifically, from a quadratical dependency when *n* = 1, we reach asymptotically a linear dependency.

For Method 2, given that the way in which the number of new rows to be assigned for every replica strongly varies from the position of the input volume patch that the kernel takes as input, we did not derive a closed formula expression. We note however that as Method 2 parallelizes computations across both directions of the (*H, W*) plane of the input volume, the theoretical minimum aspect ratio is:

(3) $$\begin{array}{l} A\left(n=H_{out}\cdot W_{out}\right)=\frac{C_{in}\cdot H_{in}\cdot W_{in}}{C_{out}\cdot H_{out}\cdot W_{out}} \end{array}$$

which, for layers with *C*_*in*_ = *C*_*out*_, *H*_*in*_ = *H*_*out*_, *W*_*in*_ = *W*_*out*_ and stride equal to 1, is equal to 1.

**Figure 6:**
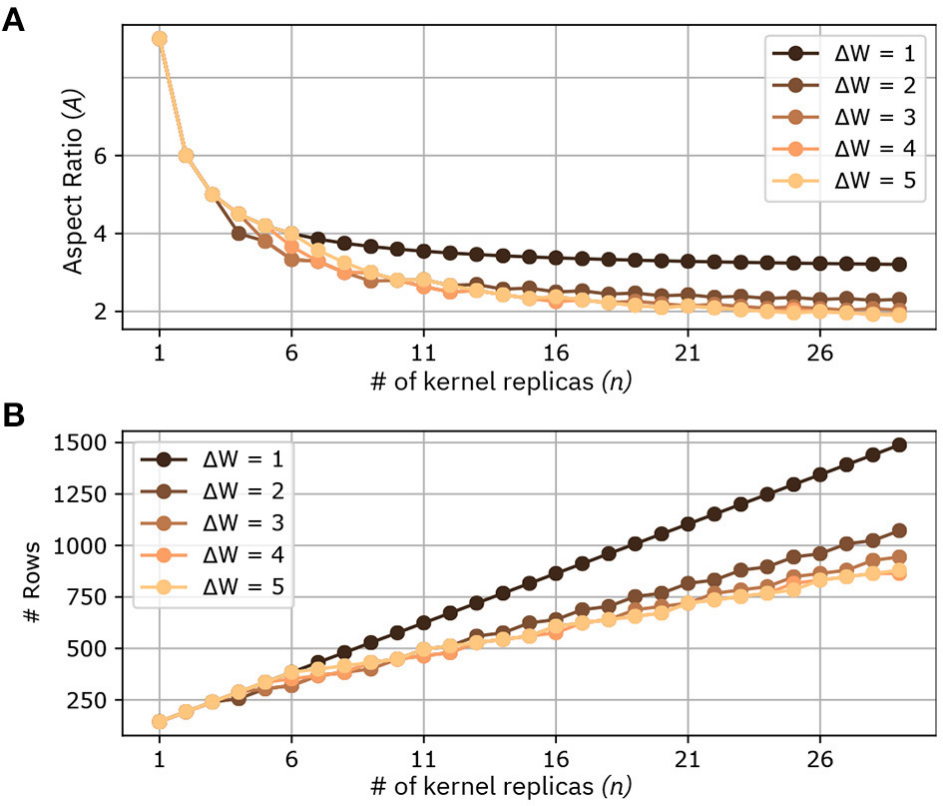
Comparison between different mappings of kernel replicas at the increase of the number of kernels *n*. Different methods are marked by different Δ*W*. **(A)** Aspect ratio of the collective kernel matrix (# crossbar rows/# crossbar columns) for a given number of kernel replicas. **(B)** Number of Rows at the increase of the number of kernel replicas.

Figure 6 shows at comparison of the two methods for different parameters. In this comparison we consider *C*_*in*_ = *C*_*out*_ = 16 and *F*_1_ = *F*_2_ = *F* = 3, which are realistic parameters for CNNs targeting IoT-like datasets. We perform the comparison in the following way: given an output volume, we fix a maximum number of pixels on the width direction *W*_*out*_ in its (*H, W*) plane (In Figure 6 it is called Δ*W*). We then parallelize the computations in order to obtain adjacent pixels with maximum width equal to Δ*W*. By comparing the two methods in this way, we observe that the case with Δ*W* = 1 corresponds to Method 1, while any other value of Δ*W* corresponds to Method 2. Figure 6A shows a comparison of the aspect ratios of the collective kernel matrix as a function of the number of kernel replicas *n*. As foreseen by Equation 2, the minimum aspect ratio reachable with Δ*W* = 1 is 3. Method 2, with (Δ*W* ≥ 2) is able to further reduce the aspect ratio, and while the theoretical minimum is equal to 1, it tends to decrease very little at around *A* = 2. Also, it is apparent that for Δ*W* ≥ 3 the parallelization seems to perform similarly. Figure 6B shows the number of rows required for a given Δ*W*, as function of the number n of kernel replicas. For a given number of replicas, Method 2, with Δ*W* ≥ 2 clearly requires less rows compared to Method 1. For example, note that a collective kernel matrix of 20 kernels requires 1,056 rows with Method 1, and only 672 with Method 2 and Δ*W* = 5. Lastly, we discuss the benefits of our methodologies on device utilization. Mathematically, device utilization *u* can be formulated as:

(4) $$\begin{array}{l} u=\frac{\sum_{i=0}^{N-1}D_{K}}{D_{A}}, \end{array}$$

where *D*_*K*_ denotes the number of devices required for one kernel matrix, *N* represents the number of kernels, and *D*_*A*_ indicates the number of devices of the crossbar array. As our proposed mapping methods maximize *N*, they also enable higher device utilization, which increases linearly with *N*. However, note that *u* is not a function of Δ*W*. Consequently, for a given number *N* of kernel matrices that are mapped on the array, the device utilization is the same, regardless of the Δ*W* used in the mapping.

### 2.3. Mapping of Activations

As presented in section 2.1, the activations for one layer are stored on the Input Memory of the IMC core. The purpose of the Input Memory is to cache the pixels, which will serve as the input vectors for the matrix-vector multiplications that will be executed on the crossbar array. We start by describing the pattern by which pixels need to be stored and fetched from the input memory. At every timestep, if the conditions for computation are met, each IMC core must fetch a given number of pixels and provide them to the crossbar array interface for computation of the matrix-vector multiplications. Further, the IMC core may have to store the pixels that it receives from other IMC cores. We assume that the Input Memory is a static random-access memory (SRAM), which is commonly used to the end of caching operands in ASICs (Shafiee et al., 2016).

In general, for the pipelined operation of layers with stride equal to 1, at every timestep a IMC core must fetch a number of pixels equal to the kernel size and store one pixel received from a previous layer (IMC core). We note the difference between the logical placement of pixels, which are part of the input volume of pixels as shown in Figure 2, and their physical instantiation in the local memory. Given that the fetching pattern is tied to the logical placement of the pixels in the input volume, in principle we would prefer to store pixels in the Input Memory in a way that mimics their logical position. Unfortunately, this task poses several problems. First, for a single network, different layers can typically have different channel depth, i.e., pixels for different layers are represented by a different number of bits. Moreover, the channel depth can vary from network to network. On the other hand, memory arrays such as on-chip SRAM, have fixed physical structures organized in words, each word comprising a given number of bits. The overall number of words is defined as the word depth. Below, we present three methods for mapping the varying and flexible data structure of pixels onto the fixed and predetermined memory array. We also present appropriate metrics for evaluating these methods.

#### 2.3.1. Memory Mapping Evaluation Metrics

In standard on-chip memories, the granularity for fetching the data is the memory word. This means that in one clock cycle, the memory can be accessed one word at a time. The number of cycles required to store (i.e., write) and fetch (i.e., read) one pixel depends on the size of the memory word and of the pixel itself. Furthermore, in the case where different pixels are stored in a single word, the writing operation is possible through write-masks, which are a common feature in SRAMs. On the other hand, the reading operation will require bit-slicing logic to dissect the word and extract the logical units required. Although possible, both the reading and the writing of different data in the same word require the accounting of the bit-line index where one data ends and another begins.

In the following subsections, we use as a metric the number of cycles required to write one pixel and to read one kernel-row of pixels. Moreover, we consider the requirement of storing bit-line indexes for reading and writing data. Lastly, we assess the amount of memory needed to store the same number of data by the various methods.

#### 2.3.2. Intra-Word Adjacent Placing (IWAP)

**Figure 7:**
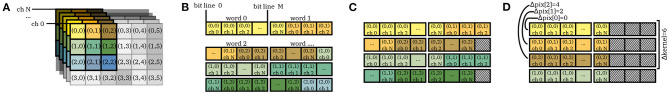
Mapping of pixels on the Input Memory. In the memory mapping, colored-in boxes represent data, while diagonal patterned boxes represent empty bitlines. **(A)** Portion of a feature map volume, in which we highlight the pixels to be mapped on the Input Memory. **(B)** Inter-word adjacent placing of pixels. In the figure, we display the numbering of words and bit lines in the Input Memory. **(C)** Kernel-level interleaved placing. Pixels belonging to one kernel row are placed contiguously to each other [e.g., pixels (0, 0) to (0, 2))]; the remaining bit lines for the word containing the (0, 2) pixel as left empty. **(D)** Activation-level interleaved placing. No data from different pixels in stored in the same word. Note that Δ*pix*[*i*] indicates the index of word with the beginning of the *i* − *th* pixel. Δ*kernel* denotes the overall number of words that store one row of pixels.

Using this method, we store pixels adjacent to each other in the memory so that different pixels could be stored on the same word. The placing mimics the logical organization of the data, so that adjacent pixels on the same row in the input volume are stored adjacent to each other on the Input Memory. Figure 7B shows inter-word adjacent mapping for a number of pixels highlighted in Figure 7A. Each pixel is mapped so that different channels are stored sequentially and according to their logical order in the feature map volume. If space is available within one word, different pixels can be stored in the same word; this is for example the case of pixel (0, 0) and pixel (0, 1) in word 1. For a given number of pixels to be cached, this method requires the minimum possible amount of memory. However, it requires bit-line indexes for both storing and fetching the pixels, and can require different number of cycles for writing and reading the pixels depending on the timestep.

#### 2.3.3. Kernel-Level Interleaved Placing (KLIP)

Using this method, we store different pixels within the same word, only if they belong to a single row of an input patch for which matrix-vector multiplications must be computed. Assume that, in Figure 7A, the feature map volume is to be convolved with a 3x3 kernel. Among those highlighted in the figure, the rows of pixels belonging to input patches for the convolution are [(0, 0), (0, 1), (0, 2)]; [(1, 0), (1, 1), (1, 2)]; and [(2, 0), (2, 1), (2, 2)]. Figure 7C shows the mapping on the Input Memory. The pixels of each row are stored contiguously to each other in the memory. However, the remaining bit lines within the word containing the last pixel of the feature map volume are left empty. This practice of leaving part of the memory empty in order to allow the mapping of data in a way that is closer to its logical positioning is somewhat similar to the concept of memory interleaving typically employed in memory management for CPUs.

As this method leaves part of the memory empty to accommodate the positioning of pixels in memory, it clearly does not use the minimum amount of memory possible for one volume of pixels. As with the previous method, it may require different number of cycles for writing the pixels depending on the timestep. Moreover, given that different pixels can still be stored within one word, bit line indexes are required for writing. As described in Figure 2, at every timestep where computations are executed, a new row of an input patch is loaded in the memory. Since we place contiguously the pixels of the rows of the input patches, the number of read cycles is constant at every timestep, and no bit line index is required for reading one row.

#### 2.3.4. Pixel-Level Interleaved Placing (PLIP)

Using this method, we do not allow storing different pixels within the same word. Figure 7D shows one such memory mapping. In the example in Figure 7A, each pixel is stored in two memory words. The unoccupied part of the last word storing each pixel is left empty. This is very similar to the concept of kernel-level interleaved placing, this time applied to single pixels across the channel depth.

This method has the finest granularity of logical units to be stored, and thus makes the most inefficient use of the local memory. Since every pixel is individually interleaved, there is no need for bit line index for either writing or storing words. Also contrary to previous methods, both the number of read and write cycles are constant regardless of the pixels being read or stored.

#### 2.3.5. Comparison of the Methodologies

In this subsection, we compare the various memory-mapping methodologies, discussed above, using the metrics introduced in section 2.3.1. Consider the storing of a portion of the feature map volume of size *H* · *F* · *N*, where *H* denotes the height of the volume, *F* the kernel size of that layer and *N* the channel depth. For the cases considered in this comparison, we use a fixed kernel size *F* = 3, whereas *H* and *N* are varying parameters. Furthermore, for this comparison we consider 8-bit precision for the pixels. As explained in section 2.3.1, at each cycle one pixel across all channels must be written on the Input Memory, while a number of pixels equal to one row of input patch must be fetched to execute the matrix-vector multiplication. Thus, in our comparison, write cycles refers to the number of cycles to write one pixel, while read cycles refers to the number of cycles to read *F* = 3 pixels.

Table 1 shows the results for various memory mappings. Firstly, we investigate the case of writing and reading the input image of a CNN, which typically comprises *N* = 3 channels. The size of the image is based on input images for the CIFAR-10 dataset, i.e., *H* = 32. Word length (*WL*) is set to 128 bits. It can be noted how inter-word adjacent placing (IWAP)requires the minimum amount of memory, but has a varying number of read and write cycles based on the pixels to be read. In this case, owing to the small size of the pixels in comparison to the word length, kernel-level interleaved placing (KLIP) and Activation-level interleaved placing (PLIP) cause a severe overhead of empty memory because of interleaving, i.e., 43 and 82.1%, respectively.

**Table 1:** Memory mapping metrics for different channel depth of the pixels (in table, *N*) and word length (*WL*).

		**Memory [KB]**	**% Empty**	**# Read**	**# Write**
**N = 3**	IWAP	0.288	0	[1, 2]	[1, 2]
**H = 32**	KLIP	0.505	43	1	1
**WL = 128**	PLIP	1.523	82.1	3	1
**N = 56**	IWAP	1.344	0	[9, 10]	[3, 4]
**H = 8**	KLIP	1.427	5.9	9	[3, 4]
**WL = 160**	PLIP	1.436	6.4	9	3
**N = 56**	IWAP	1.344	0	11	4
**H = 8**	KLIP	1.4	4	11	4
**WL = 128**	PLIP	1.528	12	12	4

*For all cases, we consider 8-bit precision. Numbers in brackets indicate that different numbers can be obtained for reading or writing different pixels. N and H are the channel depth and the height of the volume of feature maps, respectively. WL is the word length of the local memory*.

Secondly, we look at the mapping of a volume with *N* = 56 and *H* = 8. This corresponds, for example, to the parameters of the last set of layers of the ResNet-32 architecture with some channels being pruned, as in Joshi et al. (2020). For *WL* = 160, IWAP has varying read and write cycle counts. KLIP guarantees constant read cycle count, but the write cycles can still vary between 3 and 4 based on the pixel to be written, with a small overhead of empty memory, which is about 6%. PLIP guarantees a constant read and write cycle time, with an almost identical empty memory overhead in the case of KLIP. Note that PLIP also guarantees the minimum number of read and write cycles compared to both IWAP and KLIP.

Lastly, we look at the mapping with of a volume with *N* = 56 and *H* = 8, but with *WL* = 128. While IWAP does not guarantee constant read and write cycle count, in this scenario it outperforms all other methods by providing constant and minimum read and write count, other than of course minimum memory requirements. This can be accounted for by the fact that the number of bits of one pixel is exactly 3.5 times the word length. KLIP performs identically to IWAP on the metrics considered, while adding a 4% empty memory overhead. Lastly, PLIP performs the worst by requiring one additional read more compared to the other two methods, and 12% memory overhead. The comparison gives evidence of how different methodologies perform differently for different pixel sizes and word length of the memory, and no methodology outperforms the others a priori. In general terms, IWAP and KLIP perform better for pixels that have a small size compared to the word length, but require the use of write masks to store pixels and, once read, post processing for separating the bits in the word that are required from those that are unwanted. Also, the fact that neither method guarantees a constant number of reading and writing cycles, may be problematic to the design of the dataflow. Conversely, PLIP typically provides better performance for pixel sizes that are greater than the word length, and gives constant read and write cycles count regardless of word length and pixel size. It also does not require the use of write masks, and it may only require the use of post processing for selecting non-zero bits inside the words. These advantages come at the expense of a greater memory requirement. No method, in principle, guarantees a lower read and write cycle count. Lastly, we note that for pixel sizes that are exact multiples of the word size, the three methods map pixels and perform in the exactly the same way.

### 2.4. Dataflow and Memory Control

Contrary to von-Neumann computing paradigms, IMC considers one operand of the matrix-vector multiplication to be physically instantiated and ready for execution. In the case of inference of CNNs, the two operands of the matrix-vector multiplication are, as presented above, activations and kernel weights. Inherently to the functionality of hardware based on IMC, the kernel weights for every layer are physically instantiated on the devices of the computational memory in the IMC cores, and stationary on their assigned core during execution. This contrasts with von-Neumann hardware models, where the kernel weights for each layer have to be fetched from a memory, and would not be ready for use at the same time. Because of this, in order to increase the utilization of the IMC cores, we would like in principle to parallelize the operation across layers. CNNs are a class of neural networks particularly suited for this specific parallelization because, as detailed in the previous sections and contrary to other classes of neural networks, the matrix-vector operation per layer depends only on a subset of the overall volume of pixels produced by the previous layer or layers. In this section, we present a method to execute inference of such networks by pipelining across matrix-vector multiplications of different layers.

Assume an array of IMC cores interconnected by a communication fabric. Fundamentally, the dataflow implies that each IMC core operates independently, receiving pixels from cores interconnected to it and triggering its own computations once enough pixels have been received, according to the parameters of the layer it is executing. Upon completion of computation, it will deliver the data to the cores that require it. It is evident from this first summary of the dataflow that the execution will depend highly from the communication fabric that interconnects the IMC cores array. In this work, we assume the presence of a communication fabric that allows any IMC core to communicate with a point-to-point connection to any other IMC core that would require its data for a given application. One such communication fabric and the principles by which to organize communication between an array of IMC cores for the execution of CNNs are described in depth in Dazzi et al. (2019). In our IMC core architecture, the dataflow is enforced by the Dataflow Controller, which also generates the addresses for writing and reading pixels as described in section2.3. We discuss the dataflow control in section 2.4.1, and memory control in section 2.4.2.

#### 2.4.1. Dataflow Control

Algorithm 1 provides a pseudocode that describes the activity of the Dataflow Controller. At every timestep (line 2) the Dataflow Controller first checks the presence of new incoming pixels and stores them in the Input Memory according to one pixel mapping method (lines 3 to 7). Subsequently, it also updates the pointer (*pointer*_*in*) that keeps track of the current position of the pixels in the input volume. Secondly (lines 10 to 17), it checks whether the conditions to execute the computation are satisfied. When the computation is parallelized, the operations of storing the pixel and updating the input pointers are repeated a number of times (line 4) equal to the number of pixels that are being received (*nr*_*parallel*). The conditions to start the computation will depend on the pixels present in the Input Memory (and thus from *pointer*_*in*), and on the parameters of the layer, for example on whether the layer uses padding or the stride of the convolution. In case these conditions are met, the Dataflow Controller reads the pixels from the Input Memory and makes them available to the crossbar array interface (lines 12, 13). Further, it updates a pointer of the output volume that is being computed (*pointer*_*out*), which will rise a flag indicating the completion of the computation (lines 17, 18) once all the data of the output volume had been computed. Also for the case of fetching the pixels from the Input Memory, in the case of parallelization, the operations of reading the pixels and updating the output pointers is repeated a number of times equal to the number of pixels that are being produced (line 11). Lastly, the Dataflow Controller checks whether the conditions to send the residual pixel to other IMC cores are met (lines 21, 22), and in case they are verified reads the data from the Input Memory and makes it available to the inter-core communication fabric.

**Algorithm 1:** Dataflow Control

1: **def** dataflow_ ctrl():
2: **at every** timestep **do**
3: **if** *pixel*_*in* **then**
4: **for** *i* **from** 0 **to** (*nr*_*parallel*−1) **do**
5: store_data(*pointer*_*in*, *pixel*_*in*)
6: *pointer*_*in*.*x*.update()
7: *pointer*_*in*.*y*.update()
8: **end for**
9: **end if**
10: **if** conditions_to_compute(*pointer*_*in*): **then**
11: **for** *i* **from** (*nr*_*parallel*−1) **downto** 0 **do**
12: *data*_*pixels*=read_data(*pointer*_*in*-*i*)
13: data_to_crossbar(*data*_*pixels*)
14: *pointer*_*out*.*x*.update()
15: *pointer*_*out*.*y*.update()
16: **end for**
17: **if** *pointer*_*out* == *end* **then**
18: *computation*_*complete*=True
19: **end if**
20: **end if**
21: **if** conditions_to_residual(*pointer*_*in*) **then**
22: read_res()
23: **end if**
24: **end**

#### 2.4.2. Memory Control

For the control of the Input Memory, the duty of the Dataflow Controller is to generate the read and write addresses for writing and reading the appropriate data. As discussed in section 2.3, the read and write addresses comprise the word address and may or may not include bitline addresses for write masks and bit slicing. In this section, we will consider the case of PLIP memory mapping, in which write masks are not required and the address represents only the address of the memory words. Algorithm 2 shows two functions, store_data and read_data. The store_data function is called by the Dataflow Controller each time there is new incoming pixels. In principle, one may want to store the entire volume of pixels in the Input Memory. Nevertheless, by pipelining across matrix-vector multiplications, the computation is executed on the data as soon as this is available in the Input Memory, and the input volume is never needed in its entirety for executing the computation. Namely, for a kernel of size *F*_1_ = *F*_2_ = *F* with padding *P* and an image of size *H* by *H*, given the dataflow presented above, the minimum number of pixels that need to be stored in order to start the computation is *H* · (*F* − *P* − 1) + (*F* − *P*), regardless of the stride of the layer. For the sake of simplicity of the memory mapping, at the cost of a small memory overhead, we decide to store in memory *H* · *F* pixels. Note that the choice of the number of pixels to store is simply made on grounds of an easier memory mapping, and it is independent from the memory mapping strategies described in section 2.3.

**Algorithm 2:** Memory Control

1: **def** store_data(*pointer*_*in*, *pixel*_*in*):
2: write_to_SRAM(*addr*_*w*_0, *pixel*_*in*)
3: **if** *pointer*_*in*.*x* == 0 **then**
4: *addr*_*w*_0=Δ*pix*[mod(*pointer*_*in*.*y*;*kernel*_*size*)]
5: **else**
6: *addr*_*w*_0+=Δ*kernel*
7: **end if**
8: **def** read_data(*pointer*_*in*):
9: **for** *i* = 1, …, *kernel*_*size* **do**
10: *addr*_*r*=*addr*_*r*_0+Δ*pix*·mod(*pointer*_*in*.*y*+*i*;*kernel*_*size*)
11: *data*_*out*.append(read_from_SRAM(*addr*_*r*))
12: **end for**
13: **if** *pointer*_*in*.*x* == 0 **then**
14: *addr*_*r*_0=Δ*pix*[0]
15: **else**
16: *addr*_*r*_0=Δ*kernel*
17: **end if**
18: **return** *data*_*out*

In the store_data pseudocode, at line 2, the incoming data *pixel*_*in* is written to the Input Memory starting from the cached memory address *addr*_*w*_0. For any incoming pixels within one column (lines 5 to 7), the new address is computed by incrementing the current address by a quantity Δ*kernel*, representing the number of words required to store *F* pixels. This can be seen clearly in the example in Figure 7D, where Δ*kernel* is 6 words, so that two consecutive pixels on the same column [e.g., (0, 0) and (1, 0) in Figure 7A] are stored Δ*kernel* words apart. When changing column of pixels (line 3), the address must be first reset to the first position of that column in the memory. This is done by resetting *addr*_*w*_0 to one position Δ*pix*, which stores the position of the first pixel in one column. With reference to Figure 7D, Δ*pix*[0] = 0, Δ*pix*[1] = 2, Δ*pix*[2] = 4. When storing the first pixel of column *y* = 1 in Figure 7A, i.e., pixel (0, 1), *addr*_*w*_0 must be reset to Δ*pix*[1]. Since, as described above, we want to store an *H* · *F* portion of the input volume of pixels, we must take into consideration how to write pixels in the memory once we reach a column greater than the *F* − *th* column. Again with reference to Figure 7D, when storing pixels from column *y* = 3, we would reset the initial address to Δ*pix*[0], effectively storing column *y* = 3 in place of column *y* = 0, which by that timestep would have become obsolete in terms of computation. Thus, we would effectively permute the position of the column inside the Input Memory between *F* columns. This is expressed by the mod function in line 4 of Algorithm 2.

As relates to reading back the pixels from the Input Memory, the functionality is expressed by the read_data function. With reference to Figure 2, we take now in consideration the case in which one row of pixels is read from the Input Memory (e.g., the blue row of pixels in the timestep *t*_0_ + 1 portion of Figure 1). The read_data function loops through *kernel*_*size* pixels (line 9) and generates the addresses from which every pixel starts to be read by the read_from_SRAM function (line 11). The various pixels are appended to one another (line 11) and ultimately returned by the function (line 14). The reordering of the pixels because of the permutation of their positions in the Input Memory that was dealt with in the description of the store_data function is performed during the generation of the addresses. Starting from one address indicating the beginning of a row of contiguous pixels *addr*_*r*_0, this is incremented in the reading loop in the order defined by the current permutation of the positions of the pixels. Taking again as an example the storing of the pixels in Figure 7A as shown in Figure 7D, in the case in which column *y* = 3 was the last column to be stored in the Input Memory, the ordering of adjacent pixels in memory would be column *y* = 3, column *y* = 1, and column *y* = 2. Thus, for the first row of pixels, the order would be (0, 3) in words 0 and 1; (0, 1) in words 2 and 3; (0, 2) in words 4 and 5. As expressed by the mod function in line 10, starting in this case from *addr*_*r*_0 = 0, *addr*_*r* is incremented during the loop so that the pixels are read in the order (0, 1), (0, 2), (0, 3). *addr*_*r*_0 is then incremented to the initial position of the next row, or reset (lines 13 to 17). Note that, in the case of a convolution with padding, it is equivalent to having additional pixels equal to zero at the borders of the input volume. This can be addressed in two ways, either by pre-emptively storing zero pixels in the Input Memory (thus having the store_data function handle the padding) or by adding the zeros once the pixels are read from the Input Memory (thus having the read_data function handle the padding). In the next sections, we will consider the former option.

#### 2.4.3. Example of the Overall Dataflow

**Figure 8:**
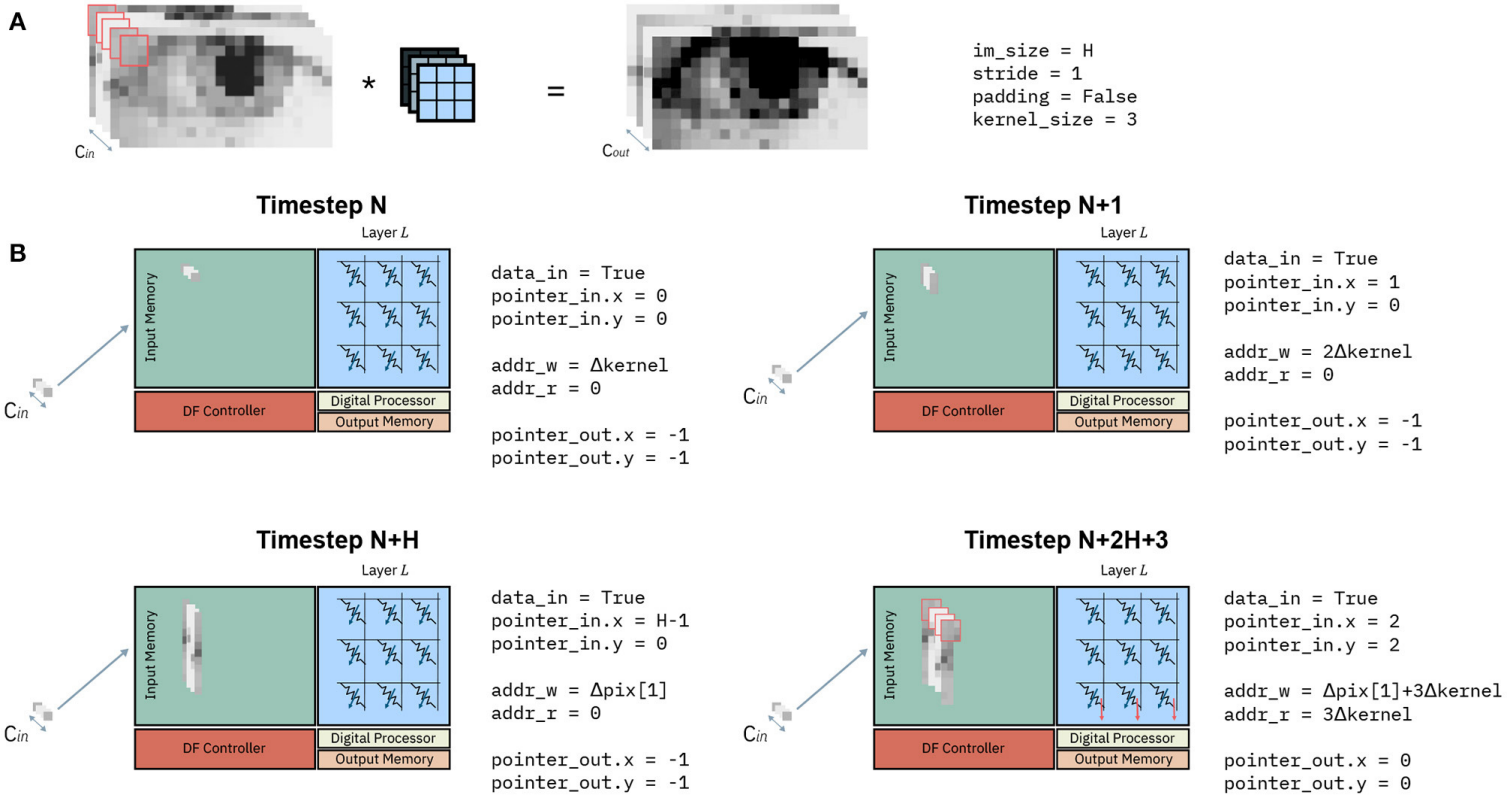
Example of dataflow for one IMC core at subsequent timesteps. **(A)** Example of a convolution. The input volume of depth *C*_*in*_ (left) is convolved with the kernel **(in blue)** to obtain an output volume of depth *C*_*out*_. **(B)** Input memory and state variables of an IMC core at different timesteps.

Figure 8 shows an example of the evolution of the state variables in Algorithm 1 at different timesteps. Consider a convolution as in Figure 8A, representing a generic layer *L* of a CNN. It transforms an input volume (left-hand side) to an output volume (right hand-side) with a kernel size of 3 × 3, stride =1 and without padding. Figure 8B shows the input memory and state variables of the dataflow control at 4 different timesteps. Before timestep N, the Input Memory is empty, the memory addresses *addr*_*w*_0 and *addr*_*r*_0 are at zero and the pointer *pointer*_*in* and *pointer*_*out* are at an invalid value (−1). At a Timestep N, the first pixel is received. This corresponds to the pixel at position (0, 0) on the (*x, y*) plane, and the pointer to the input volume are consequently updated to values *pointer*_*in*.*x* = 0, *pointer*_*in*.*y* = 0. According to the PLIP memory mapping, the initial address to store the next pixel is updated to *addr*_*w*_0 = Δ*kernel*. Lastly, since a single pixel is not sufficient to perform any computation, the conditions to compute return *False*, and *addr*_*r*_0 and *pointer*_*out* are not updated. At the subsequent timestep N+1, another pixel along the column *y* = 0 is received. Similarly to the previous timestep, *pointer*_*in* is updated to (1, 0), *addr*_*w*_0 is incremented of Δ*kernel*, while the other pointers remain as they were. At some timestep N+H, the entire column *y* = 0 has been received and store in the Input Memory. Again, the update of *pointer*_*in* reflects the position reached in the input volume, which is still not enough to perform any matrix-vector multiplication with a 3x3 kernel without padding. As one column has been received in entirety, *addr*_*w*_0 will have to be reset to the initial position of the second column with *y* = 1, which is equal to Δ*pix*[1]. Finally, 2H + 3 timesteps after the initial timestep N, two entire columns and three pixels of the third column have been received, and the input pointer *pointer*_*in* is updated to position (2, 2). As the layer executes a 3 × 3 convolution without padding, there is sufficient data to execute the first matrix-vector multiplication. The condition_to_compute at line 7 of Algorithm 1 returns *True* and the input data, highlighted in red in Figure 8, is read from the Input Memory and provided to the interface of the crossbar. This matrix-vector multiplication results in the upper leftmost pixel of the output volume, and thus the output pointer *pointer*_*out* is updated to the newly computed position (0, 0). Lastly, the initial address for data to be read is updated to the position of the latest pixel to be read, equal to 3Δ*kernel*.

#### 2.4.4. Splitting of Layers Onto Multiple IMC Cores

So far, we assumed the kernel matrix can fit in its entirety on the crossbar array of the IMC core. Nevertheless, because of the variability of kernel matrices even within a single CNN on the one hand and the fixed size of crossbar arrays on the other, we must take into consideration the case in which one crossbar array does not have enough rows or columns to fit one kernel matrix. Without loss of generality, we consider the two cases separately, one where the number of rows in one crossbar is not sufficient to fit one kernel matrix and one where the number of columns is not sufficient. As expressed in section 2.2, because of the typical shape of kernel matrices on computational memory, the former case will be, in principle, the most common.

Assume the case of a crossbar array with *R* rows and *C* column, and layer whose kernel matrix requires *R*_*KM*_ rows and *C*_*KM*_ columns. If 2*R* ≥ *R*_*KM*_ > *R* and *C*_*KM*_ ≤ *C*, we can split the kernel matrix between two crossbar arrays so that they calculate partial accumulations of the same convolution. This is equivalent to splitting the original matrix-vector multiplication between a patch of the input volume **α**_*i*:*i*+*F*−1, *j*:*j*+*F*−1_ and a kernel matrix ***K*** as in Equation (5), with each IMC core executing one matrix-vector multiplication.

(5) $$\begin{array}{l} \begin{array}{c} \alpha_{i:i+F-1,j:j+F-1}\cdot K={\left[\begin{array}{c} \alpha_{i,j}^{0}\\ \ldots \\ \alpha_{i+F-1,j+F-1}^{N/2-1}\\ \alpha_{i,j}^{N/2}\\ \ldots \\ \alpha_{i+F-1,j+F-1}^{N-1} \end{array}\right]}^{T}\left[\begin{array}{ccc} k_{0,0}^{0,0} & \ldots & k_{0,0}^{0,M}\\ \ldots & \ldots & \ldots \\ k_{F-1,F-1}^{N/2-1,0} & \ldots & k_{F-1,F-1}^{N/2-1,M}\\ k_{0,0}^{N/2,0} & \ldots & k_{0,0}^{N/2,M}\\ \ldots & \ldots & \ldots \\ k_{F-1,F-1}^{N,0} & \ldots & k_{F-1,F-1}^{N,M} \end{array}\right]=\\ \;={\left[\begin{array}{c} \alpha_{i,j}^{0}\\ \ldots \\ \alpha_{i+F-1,j+F-1}^{N/2-1} \end{array}\right]}^{T}\left[\begin{array}{ccc} k_{0,0}^{0,0} & \ldots & k_{0,0}^{0,M}\\ \ldots & \ldots & \ldots \\ k_{F-1,F-1}^{N/2-1,0} & \ldots & k_{F-1,F-1}^{N/2-1,M} \end{array}\right]+\\ \;+{\left[\begin{array}{c} \alpha_{i,j}^{N/2}\\ \ldots \\ \alpha_{i+F-1,j+F-1}^{N-1} \end{array}\right]}^{T}\left[\begin{array}{ccc} k_{0,0}^{N/2,0} & \ldots & k_{0,0}^{N/2,M}\\ \ldots & \ldots & \ldots \\ k_{F-1,F-1}^{N,0} & \ldots & k_{F-1,F-1}^{N,M} \end{array}\right] \end{array} \end{array}$$

This is effectively equivalent to splitting the layer into two different layers, where the IMC core executing the first half of the computation forwards it to the other IMC core, which sums the two partial results and execute the required digital processing (batch normalization, activation function and possibly residual additions). In terms of dataflow, this is not different from having two separate layers.

Assume now the case in which *R*_*KM*_ ≤ *R* and 2*C* ≥ *C*_*KM*_ > *C*. In this case, we can split the kernel matrix between two crossbar arrays so that they calculate different channels of the same convolution. This is equivalent to performing the splitting shown in Equation (6), where ⊕ indicates the concatenation operation. This is also equivalent to splitting the layer into two different layers, and the same considerations made for the previous case hold.

(6) $$\begin{array}{l} \begin{array}{c} \alpha_{i:i+F-1,j:j+F-1}\cdot K={\left[\begin{array}{c} \alpha_{i,j}^{0}\\ \ldots \\ \alpha_{i+F-1,j+F-1}^{N-1} \end{array}\right]}^{T}\left[\begin{array}{ccc} k_{0,0}^{0,0} & \ldots & k_{0,0}^{0,M/2-1}\\ \ldots . & \ldots . & \ldots \\ k_{F-1,F-1}^{N-1,0} & \ldots & k_{F-1,F-1}^{N-1,M/2-1} \end{array}\right]⊕\\ \;{\left[\begin{array}{c} \alpha_{i,j}^{0}\\ \ldots \\ \alpha_{i+F-1,j+F-1}^{N-1} \end{array}\right]}^{T}\left[\begin{array}{ccc} k_{0,0}^{0,M/2} & \ldots & k_{0,0}^{0,M}\\ \ldots & \ldots & \ldots \\ k_{F-1,F-1}^{N-1,M/2} & \ldots & k_{F-1,F-1}^{N-1,M-1} \end{array}\right] \end{array} \end{array}$$

This concept can be of course generalized to a splitting into an arbitrary number of IMC cores, combining both types of splitting, provided connectivity between these IMC cores exists.

## 3. Results

### 3.1. Execution of ResNet-32 on an a IMC Accelerator

In this section, we will present the employment of our proposed methodologies in the mapping of a CNN on an array of IMC cores and discuss the behavior of the overall inter- and intra-core dataflow. Specifically, we will consider the inference of ResNet-32 for the CIFAR-10 dataset, which represents a state-of-the art accuracy network for such dataset. While our methodologies are generic with respect to the IMC technology that is employed and are orthogonal to the implementation of the crossbar array and/or the precision of the matrix-vector multiplication operations, in this section we make some assumptions on the hardware. Firstly, we assume the crossbar arrays of each IMC core to have size of 256 × 256. The crossbar arrays operate in a fully parallel mode, and utilizes Phase-Change Memory (PCM) as the IMC devices. Specifically, in this implementation, each weight is mapped onto a differential pair of PCM devices. Input and output data is provided in 8-bit int fixed precision format. Regarding the Digital Processor, this implementation assumes a light digital processing element that can perform batch normalization, residual addition and ReLU. This choice is justified by the fact that the great majority of state-of-the-art CNNs, among which ResNet-32, feature these three operations on activations. Regarding the reduced precision implementation of ResNet-32, it is based on the one presented in Joshi et al. (2020). Such implementation presents a training strategy for the same hardware and numerical precision presented in this work, and reaches 93.7% accuracy on CIFAR-10. Also in this case, note that our proposed methodologies and dataflow are orthogonal to the training process and optimization of the DNNs executed on the IMC hardware.

In this section, we first present the mapping of the network on the IMC core array in section 3.1.1. In section 3.1.2, we present the mapping strategy employed for the activations, and in section 3.1.3, we give display of the dataflow and the overall performance. In section 3.1.4, we discuss the possibility of speedup of the dataflow. Lastly, in section 3.2, we present an implementation of the dataflow controller and input memory implemented in 14 nm CMOS technology.

#### 3.1.1. Mapping of the Kernel Weights on the Array

Figure 1A shows a representation of ResNet-32 and its mapping on an array of IMC cores. ResNet-32 comprises 34 layers. Specifically, 31 convolutional layers with kernel size *F*_1_ = *F*_2_ = *F* = 3, one fully connected layer at the end of the network for classification, and two layers for resampling of the residual connection with kernel size *F*_1_ = *F*_2_ = *F* = 1. Firstly, we must store the kernel matrices on the computational memory of the IMC core array. We proceed in the following way: we map one layer per IMC core; in case the crossbar array did not have enough rows or columns to fit the kernel matrix for one layer, we split the kernel matrix and map it on multiple IMC cores. For the sake of simplicity, in this first discussion of the dataflow, we will assume the mapping of a single kernel matrix per layer, that is, no kernel replication as in section 2.2. Table 2 reports the required number of rows and columns for the kernel matrices of each layer. In order to better match the size of the crossbar array, we have trained a version of ResNet-32 with a slightly lower number of channels for the layers RS1, RS2, 12, 32 and FC. In general, all channel depth of 32 have been reduced to 28, and all channel depths of 64 to 56. Such modification is consistent with the implementation in Joshi et al. (2020). Given the number of required rows and columns, all layers except 22/31 can be mapped onto a single crossbar array. Layers 22/31 require more rows i.e., 504, than the 256 rows available in a single crossbar array. Thus, for each of these layers we split the kernel matrix into two smaller matrices of size 256 × 56. In this way, we perform two partial accumulations of the overall matrix-vector multiplication. Note that, for these layers, activations α^0, *L*^ to α^27, *L*^ are input to one crossbar array, and α^28, *L*^ to α^56, *L*^ are input to another one. We will discuss in more detail, in section 3.1.3, how these two partial accumulations are combined. As a result of this overall mapping strategy, the ResNet-32 network requires 43 IMC cores.

**Table 2:** Layer specifications for ResNet-32 network.

**#Layers**	***H*_*in*_ × *W*_*in*_**	***H*_*out*_ × *W*_*out*_**	**Stride**	***C*_*in*_**	***C*_*out*_**	**Kernel Size**	**#Rows**	**#Columns**	**Pixel Size [bits]**
1	32 × 32	32 × 32	1	3	16	3 × 3	27	16	24
2/11	32 × 32	32 × 32	1	16	16	3 × 3	144	16	128
RS1	32 × 32	16 × 16	2	16	28	1 × 1	16	28	128
12	32 × 32	16 × 16	2	28	28	3 × 3	252	28	224
13/21	16 × 16	16 × 16	1	28	28	3 × 3	252	28	224
RS2	16 × 16	8 × 8	2	28	56	1 × 1	28	56	224
22	16 × 16	8 × 8	2	56	56	3 × 3	504	56	448
23/31	8 × 8	8 × 8	2	56	56	3 × 3	504	56	448
FC	1 × 1	1 × 1	2	56	10	/	56	10	448

#### 3.1.2. Mapping of Activations on the Input Memory

Table 2 shows the parameters of the layers of ResNet-32 as well as the size in bits of every pixel that needs to be stored in the Input Memory of the corresponding IMC core. We have assumed a word length of 128 bits and a word depth of 512, amounting to a total to 8KB SRAM for the Input Memory of each IMC core. We have used IWAP to store the pixels for layer 1 and ALIP for storing pixels for the remaining layers. In fact, as noted in section 2.3.5, IWAP works best for layers where the size of the pixels is small compared to the word length, as is the case with layer 1, while for the other layers KLIP or PLIP are preferable. From the consideration on the minimum memory requirements in section 2.4.2, the memory requirements for the different convolutional layers are 0.28 KB for layer 1 and 1.5 KB for all the others. Both requirements are lower than the size of the Input Memory.

#### 3.1.3. Dataflow and Performance

**Figure 9:**
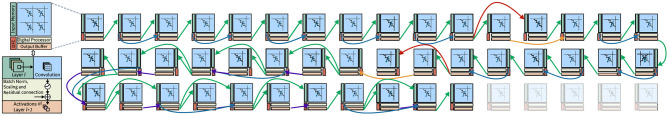
Mapping of ResNet-32 on an array of IMC cores. Different colored arrows represent different functionality of the communication. Specifically, green arrows represents feedforward communication and the blue ones denote residual connections. Moreover, the red arrows indicate communication to resampling layers, whereas the orange ones represent residual connections from a resampling layer. Finally, the purple arrows signify partial summation for layers split on multiple IMC cores.

**Figure 10:**
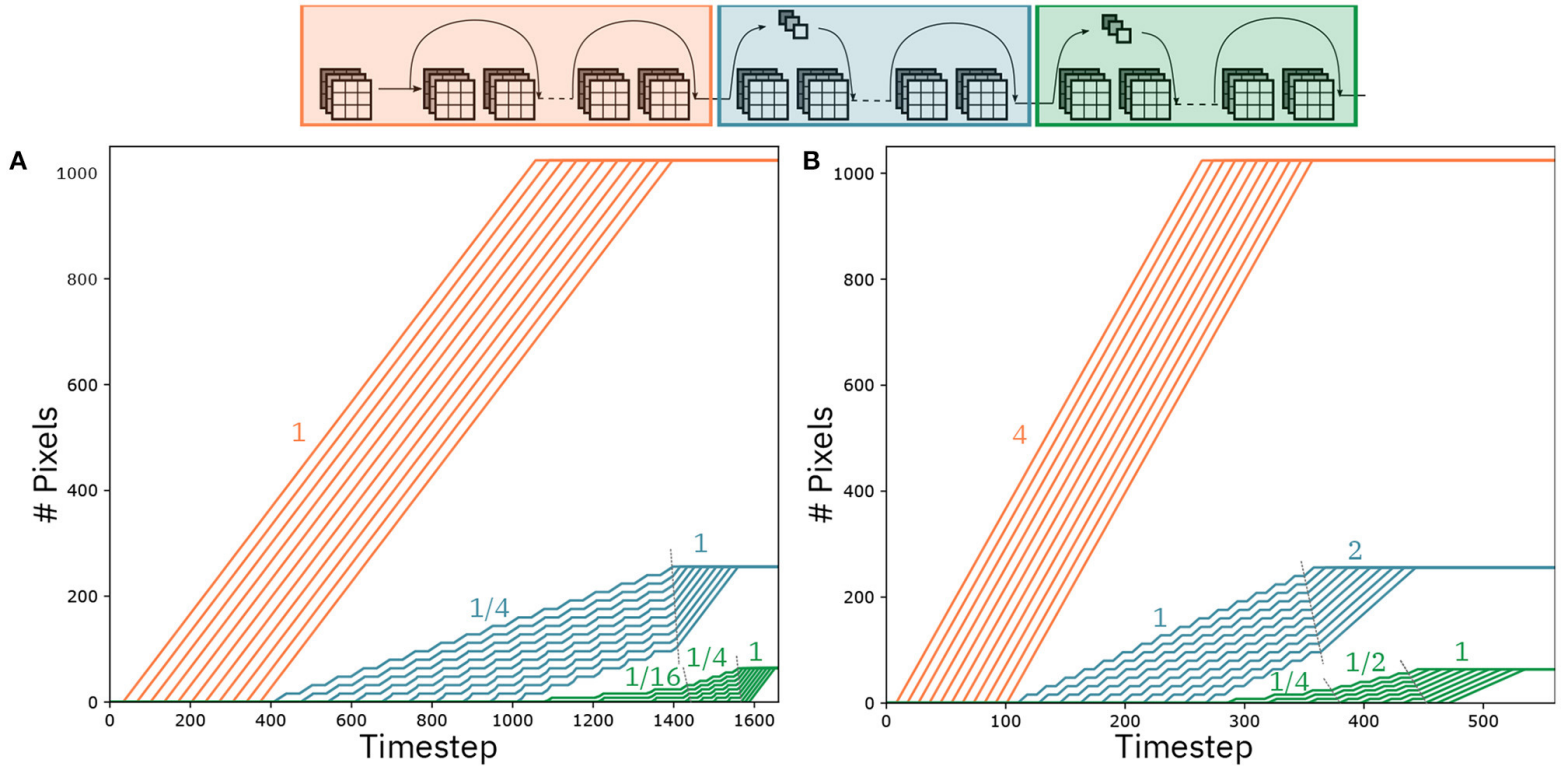
Pixels per layer as a function of the timesteps. Layers with different feature map size are color-coded in different colors. **(A)** Pixels being computed by mapping at most 1 kernel matrix per layer. **(B)** Pixels being computed by mapping multiple kernel matrices per layer.

Figure 9 shows the mapping of ResNet-32 on an 8-by-6 array of IMC cores. Arrows between cores represent communication channels. We assume a communication fabric as in Dazzi et al. (2019), such that every connection can be satisfied as required by the network. This topology assures that all data delivery occurs within one timestep, guaranteeing that the pipeline never stalls. In Figure 9 different colored arrows represent different functionality of the communication. Specifically, green arrows represents feedforward communication and the blue ones denote residual connections. Moreover, the red arrows indicate communication to resampling layers, whereas the orange ones represent residual connections from a resampling layer. Finally, the purple arrows signify partial summation for layers split on multiple IMC cores. Figure 10A shows the evolution of the computation per convolutional layer as a function of the timestep. In an ideal pipelining scenario, one would expect that one pixel is computed at every timestep, such that the number of pixels to be computed per layer is equal to the number of timesteps the IMC core is active. This implies that the slope of the curves in Figure 10A should be equal to one. The slope is indeed equal to one for all layers that do not need strided convolutions (see red curves in Figure 10A). However, the computation of layer 12, with stride equal to two, can only be executed every two rows and every two columns. As the size of the feature map for that layer is 16x16, the IMC core assigned to layer 12 will perform computations every 2 timesteps, while receiving the pixels from layer 11, and stall for 16 timesteps when receiving columns on which no computation is required. Overall, on average, this implies that there will be a computation every 4 cycles, meaning the average slope is 1/4. As all the layers subsequent to layer 12 are connected in a feedforward way, the reduced slope of the computation vs. timesteps curve propagates to all the subsequent layers until the point at which layer 12 is completed. This is evident in Figure 10A, where the layers from 13 to 21 progress also at slope 1/4 up to point at which layer 12 is completed, after which the slope progresses at slope 1. Moreover, this is propagated to the layers from 22 to 31 where the slope, which is supposed to be equal to 1/4 (the stride equal to two in layer 22), is reduced of an additional factor 4, i.e., 1/16.

As a result, the overall latency for a single classification is 1,628 cycles. Assuming a timestep of 100 ns, this results in 161.8 us latency for the classification of a single image and 9,650 Images/s throughput.

#### 3.1.4. Dataflow Speedup

In an architecture as shown in Figure 9, the bandwidth of the communication links is tailored on the most expensive data communication. The bandwidth requirements are calculated as

(7) $$\begin{array}{l} B=\left(n_{\text{bits}}\cdot C_{out}^{max}\right)/T_{timestep} \end{array}$$

where *n*_bits_ denotes the number of bits per single activation, $C_{out}^{max}$ indicates the maximum number of output channels of the convolutional layers throughout the network, and *T*_*timestep*_ represents the duration in seconds of one timestep. Assuming that we want to deliver all the data from one IMC core at most within one timestep of 100 ns, the maximum bandwidth required is 4.5 Gbps, from layers 22 to 31. This corresponds to the layers colored in green in Figure 10. In ResNet-32 and generally in the design of CNNs, the number of channels per layer doubles for every group of layers. In Figure 10, the group of layers marked in blue have twice the number of channels compared to the layers in red, and the layers marked in green have four time the number of channels compared to the layers in red. From Equation 7, it follows that the same bandwidth that allows to send 1 activation in 1 timestep for the last group of layers, allows to deliver in 1 timestep 4 activations for the first group of layers and 2 for the second, respectively. Based on this observation, we present a pipeline speedup method that uses the same hardware communication requirements as the regular dataflow. Figure 10B shows the number of activations computed per layer as the timesteps increase, assuming a sped up dataflow. According to this dataflow, we assume that the IMC cores parallelize the computation of matrix-vector operations based on one of the methods presented in section 2.2. Specifically, the first group of layers (red) computes four activations per timestep, the second group of layers (blue) computes two activations per timestep, and the third computes one activation per timestep. As argued before, as long as the kernel matrices can fit on the crossbar arrays of the IMC cores, this does not modify the bandwidth requirements of the hardware implementation. In accordance with the activations per timesteps that are computed per layer, Figure 10B shows that the slope of the activation/timestep curve is equal to four for the first group of layers. This seed-up is reflected on the post-stride layers (in blue), where the slope of the layers is increased by 4x compared to Figure 10A, as long as its computation depends on the data that is received from the first set of layers, after which it proceeds at the speed set by the IMC core (2 activations/timestep). Similarly for the third group of layers, the speed-up of the preceding layers reflects on its own execution that is sped up as long as it depends on the data that are received by the previous layers, with the slope gradually increasing from 1/4 to 1/2 and ultimately to 1 when the second group of layers have finished the computations. As the number of activations that are produced per layer depend on both the rate of the preceding layers and its own rate, the speed-up is clearly non-linear. For the case of ResNet-32, it results in a latency for a single classification of 526 cycles, resulting to a speed-up of a factor 3.1. The throughput, which depends primarily on the rate of the first layers, is equal to 38,600 Images/s for a batch of 100 images to be classified.

### 3.2. Hardware Implementation

We demonstrated the dataflow presented in this work via experiments and simulation. Specifically, the Input Memory, Dataflow Controller and communication fabric have been implemented in CMOS 14nm technology. The communication fabric implements the topology presented in Dazzi et al. (2019). These elements represent the digital framework around an array of 8-by-8 IMC cores, constituting an IMC based inference engine. This architecture allows the execution of inference for CNNs through the dataflow described in section 2.4. Note that, in this implementation, we are agnostic of the computational elements, i.e., Crossbar Array and Digital Processor. Based on the application and the desired accuracy, different computational elements can be utilized in such an architecture to build the overall accelerator.

**Figure 11:**
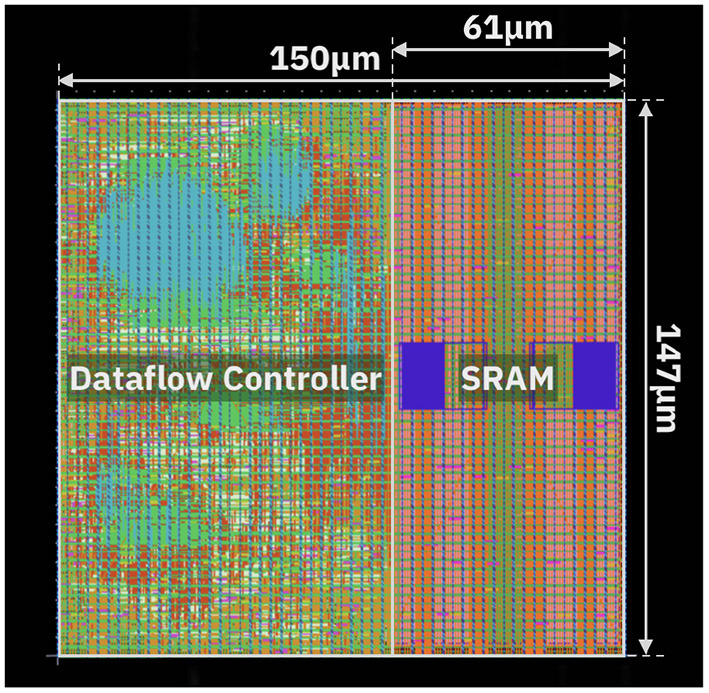
Physical layout of the Dataflow Controller and Input Memory.

The design assumes supply voltage equal to 0.8 V and clock frequency of 500 MHz. Table 3 shows the power consumption and area occupation of the blocks. The Input Memory has been implemented as an SRAM memory with word length equal to 128 bits, comprising 8 KB of memory. Figure 11 displays the layout of the Input Memory and Dataflow Controller. Overall, the Input Memory occupies 0.0091 mm^2^, while the Dataflow Controller occupies 0.0132 mm^2^. In order to have a realistic estimation of the power consumption, we evaluate the joint operation of both blocks. Specifically, storing and reading from the Input Memory is only executed based on the dataflow set by the Dataflow Controller. The joint power consumption of Dataflow Controller and Input Memory is equal to 10.54 mW. A breakdown of the power consumption is reported in Table 4. Most power is spent in the Sequential elements, which is justified considering the significant amount of buffering of data and pointers inside the Dataflow Controller.

**Table 3:** Power consumption and area occupation for Input Memory, Dataflow Controller, and communication links implemented in 14 nm CMOS technology.

**Area DFC [*mm*^2^]**	**Area SRAM [*mm*^2^]**
0.0132	0.0091
**Power DFC+SRAM [*mW*]**	**Power links [mW]**
10.544	0.775

**Table 4:** Power breakdown for the internal components of the Dataflow Controller.

	**Internal power**	**Switching Power**	**Leakage power**	**Total Power**	**Percentage (%)**
**Sequential**	4.052	0.3429	0.06095	4.455	42.25
**SRAM macro**	2.548	0.03460	0.02155	2.604	24.7
**Combinational**	0.5563	1.625	0.04357	2.225	21.1
**Clock**	0.1884	1.071	0.001539	1.261	11.96
**Total**	7.344	3.073	0.1276	10.54	100

As relates to the communication fabric, the design implements synchronous communication with latch-to-latch links. The average power consumption is 0.775 mW. The energy efficiency of the links depends on their lengths, and ranges between 39.4 and 346.5 fJ/bit.

We now discuss the usage of the computational elements within the architecture described above. In accordance with the case study presented in section 3.1, we assume crossbar arrays of size 256 × 256. We assume the use of phase-change memory (PCM) (Burr et al., 2017) as the computational memory. In order to estimate the performance of such an array, we take into consideration the analog and digital contributions to the energy consumption of the crossbar array. Specifically, as regards the analog contributions, we consider the energy for the voltage regulators for the columns of the array, the energy for the analog-domain computation and the energy of the ADCs for the analog-to-digital conversion. For the analog computation we assume the average conductance of the PCM devices to be 2.5 μS and the read voltage to be 0.2 V. Also, the energy for the ADCs is taken from Kull et al. (2017). As regards the digital contributions, for the crossbar array we also take into consideration the pulse-width modulator (PWM). The PWM applies the input to the array as voltage pulses, performing a digital-to-analog conversion from an 8-bit signed integer to a read voltage pulse. Lastly, we assume a Digital Processor performing batch normalization, ReLU and addition for the residual connection. The power consumption of the digital blocks was obtained from RTL simulations of the HDL code of one implementation. Overall, the resulting energy efficiency is 10.5 TOPS/W. Note that, although our chip design is based on 256 × 256 crossbar arrays, it can be readily estimated that by increasing the crossbar array size to 512 × 512 the efficiency becomes 16.3 TOPS/W. Comparing the resulting energy efficiency with digital accelerators targeting similar datasets (Sim et al., 2016) and IMC-based accelerators (Ando et al., 2017), our architecture outperforms them up to a factor 7.4x and 5.2x, respectively. Moreover, our implementation yields very high operations per second, providing 1.008 TOPS. Such performance is, respectively, 7.36x and 409x higher compared to SRAM-based (Jia et al., 2020) and ReRAM-based (Xue et al., 2020) IMC array implementations. In terms of performance density, our proposed PCM-based array provides 1.59 TOPS/mm^2^, outperforming the aforementioned implementations by 41.76x and 1322x, respectively. Lastly, comparing our results in terms of thoughput with the ones reported by systolic array-based ASICs on a ResNet of similar depth (Andri et al., 2018), we achieve a throughput that is 206x higher with the dataflow described in section 3.1.3 and 826x higher with the dataflow speedup method described in section 3.1.4. Furthermore, our presented throughputs are, respectively, 7x and 31x higher compared to that of ASICs targeting the same dataset (Esser et al., 2016).

## 4. Discussion

Various optimized dataflows for efficient hardware deployment of CNNs have been explored in literature. Specifically, pipelined dataflows that exploit parallelization of the computation between layers, have demonstrated mitigation of bandwidth limitations (Goetschalckx and Verhelst, 2019). Similarly, dataflows exploiting the local computation of feature maps between CNN layers have already been employed in a variety of hardware accelerators. For example, dataflows that fuse the computation between subsets of subsequent layers within a CNN (Alwani et al., 2016) have been used in implementations of CNNs on FPGA-based accelerators (Reggiani et al., 2019). Moreover, such advanced dataflows have been proposed for heterogeneous architectures in order to improve throughput and avoid use of off-chip memory (Wei et al., 2018). Dataflows that pipeline the computation across layers in an end-to-end fashion have recently been employed in accelerators based on IMC for inference (Shafiee et al., 2016), and similar concepts have also been used for training dataflows (Song et al., 2017).

All these contributions consider simple feed-forward networks. Furthermore, in all the prior works, the optimization and possible parallelization of the computation performed on the crossbar array has not been considered. In this work we present a fully pipelined, end-to-end inference dataflow based on the proposed optimized mapping of synaptic weights on the crossbars and of activations on the on-chip memory. The dataflow is agnostic of the connectivity of the CNN layers and is not limited to simple feedforward networks. Among the contributions to the mapping of kernel weights on computational memory, it is worth mentioning the results on different ordering strategies presented in Yue et al. (2020). While this work targets easier data handling in the case the kernel matrices were split onto multiple crossbars, we take an orthogonal approach by trying to optimize device utilization when mapping multiple replicas of the kernel weights on the same crossbar array. Clearly, different ordering techniques can in principle be used alongside the optimization strategies presented in this paper. Optimization of the kernel mapping has also been explored in Peng et al. (2019). This work reorders and partitions kernel matrices in order to facilitate their mapping on the crossbar arrays, and proposes a novel dataflow in order to allow DNN inference with such a kernel partition. In our work, we propose kernel mapping methods that do not require *ad-hoc* dataflows for their execution, so that they can be applied to well-established dataflows and compatible with widely used environments for neural network definitions.

To summarize, in this paper we have introduced various methodologies for accelerating convolutional neural networks (CNNs) on hardware based on IMC. We introduced an architecture of a computational memory (IMC) core for execution of CNNs. We presented an exhaustive set of methods for mapping kernel matrices on the crossbar arrays of the IMC cores. For a given number of matrix-vector multiplications to be executed in parallel, these methods allow the size of the crossbar array required to map the kernel matrices to be minimized. Secondly, we presented three novel methods for storing activations on the local memory of the IMC cores. Based on the overall size of the activations and on the parameters of the local memory used for storing them, these different methods for storing activations can minimize the number of read and write cycles required. Using these mapping schemes for both kernel matrices and activations, we then proposed an end-to-end dataflow for execution of CNNs on an array of IMC cores. We studied the performance of this dataflow on ResNet-32 for CIFAR-10 and presented the implementation of the dataflow control logic in 14nm CMOS technology, demonstrating a throughput that is at least 7x higher than achieved by ASICs targeting the same neural network and dataset.

## Data Availability Statement

Some of the datasets presented in this article are not readily available because simulators used for some results are proprietary. Other datasets presented in this study can be found in online repositories. CIFAR-10 dataset is available for download at https://www.cs.toronto.edu/~kriz/cifar.html. ResNet models are typically available in machine learning frameworks such as PyTorch (https://pytorch.org/hub/pytorch_vision_resnet/).

## Author Contributions

MD conceived the IMC core architecture with support from AS and EE. MD conceived the weight and activation mapping methodologies and developed the python simulator for the experiments. MD developed the dataflow concept for CNNs on IMC with support from AS, LB, and EE. MD designed the hardware implementation of the dataflow controller. MD and EE wrote the manuscript with inputs from LB and AS. AS, LB, and EE supervised the project. All authors contributed to the article and approved the submitted version.

## Conflict of Interest

MD, AS, and EE were employed by the company IBM Research Zurich. The remaining author declares that the research was conducted in the absence of any commercial or financial relationships that could be construed as a potential conflict of interest.

## Publisher's Note

All claims expressed in this article are solely those of the authors and do not necessarily represent those of their affiliated organizations, or those of the publisher, the editors and the reviewers. Any product that may be evaluated in this article, or claim that may be made by its manufacturer, is not guaranteed or endorsed by the publisher.
